# [Retracted] miR‑29b enhances the proliferation and migration of bone marrow mesenchymal stem cells in rats with castration‑induced osteoporosis through the PI3K/AKT and TGF‑β/Smad signaling pathways

**DOI:** 10.3892/etm.2022.11644

**Published:** 2022-10-05

**Authors:** Yuhui Wang, Xiangmin Han, Tongxin Zang, Ping Kang, Wei Jiang, Niu Niu

Exp Ther Med 20:3185–3195, 2020; DOI: 10.3892/etm.2020.9045

Following the publication of this paper, it was drawn to the Editors’ attention by a concerned reader that certain of the cell invasion assay data shown in Fig. 3D were strikingly similar to data appearing in different form in another article by different authors. Owing to the fact that the contentious data in the above article had already been published elsewhere prior to its submission to *Experimental and Therapeutic Medicine,* the Editor has decided that this paper should be retracted from the Journal. The authors were asked for an explanation to account for these concerns, but the Editorial Office did not receive a reply. The Editor apologizes to the readership for any inconvenience caused.

